# The spatial relationship between tuberculosis and alcohol outlets in the township of Mamelodi, South Africa

**DOI:** 10.4314/ahs.v22i2.19

**Published:** 2022-06

**Authors:** Lazola Booi, Gregory D Breetzke

**Affiliations:** Department of Geography, Geoinformatics and Meteorology, University of Pretoria, Pretoria, 0002, South Africa

**Keywords:** Tuberculosis and alcohol outlets, Mamelodi, South Africa

## Abstract

**Background:**

The availability of alcohol in society is known to increase the risk of a range of negative health outcomes.

**Objectives:**

The aim of this research is to determine if there is a spatial association between tuberculosis and alcohol outlets in Mamelodi, South Africa. We also aim to examine whether the socio-economic characteristics of the neighbourhood in which an alcohol outlet was located was related to the magnitude of tuberculosis in the immediate vicinity of the alcohol outlet.

**Methods:**

Location quotient analysis is used to compare the extent of tuberculosis within a series of buffer intervals (100m, 200m, 300m) immediately surrounding alcohol outlets with tuberculosis across the township of Mamelodi as a whole.

**Results:**

The density of tuberculosis around alcohol outlets in Mamelodi at all buffer distances was found to be substantially higher than across the township as a whole. These findings indicate that the risk of tuberculosis in Mamelodi is higher around alcohol outlets. Alcohol outlets located in more deprived areas of Mamelodi were significantly associated with higher density of tuberculosis relative to alcohol outlets located in more affluent neighbourhoods.

**Conclusion:**

Despite alcohol outlets forming an integral part of the urban landscape in townships in South Africa, they may facilitate the transmission of tuberculosis.

## Introduction

Tuberculosis is a preventable and curable disease that affects roughly one-third of the global population^1^. African countries are, however, disproportionately affected with tuberculosis rates well above the global average. Within Africa, South Africa has the highest annual incidence of tuberculosis with a rate of approximately 520 incidents per 100,000 population^1^. The reason for the high rates of tuberculosis in South Africa are myriad and have previously been attributed to factors such as poverty, pollution, overcrowding, malnutrition, the remoteness of health facilities, poor health care programmes and the co-occurrence with other diseases such HIV/AIDS^2^–^7^.

Although some research has been done examining the risk factors associated with tuberculosis in South Africa, very little is known how the prevalence of the disease in the country is impacted by the availability of alcohol. Alcohol usage has been identified as one of the key risk factors for a number of communicable diseases including tuberculosis 8–9, yet the specific spatial linkage between tuberculosis and the presence of alcohol outlets in South Africa has yet to be empirically determined. The main aim of this research is to determine if there is a spatial association between tuberculosis and alcohol outlets in one township community in the city of Tshwane of South Africa, namely Mamelodi. Specifically, we are interested in examining whether tuberculosis spatially clusters around alcohol outlets in the township and aim to determine whether the underlying socio-demographics of the neighbourhood in which the alcohol outlet occurs influences this association. We use point level tuberculosis data and a novel geospatial technique to examine the diffusion of tuberculosis around alcohol outlets in this unique post-apartheid setting.

## Risk factors for tuberculosis

Similar to international studies, factors influencing tuberculosis prevalence in South Africa span the social, economic, health/lifestyle and built environments. In terms of the social environment common risk factors include overcrowding^10^–^11^, roof leakage^12^, air pollution^13^, and smoking^14^, among numerous others. From an economic perspective, the poor^5^, deprived^15^, and unemployed^16^ are at a greater risk of tuberculosis in South Africa as well as those with the least access to healthcare facilities^17^. Indeed, the provision of adequate healthcare facilities in South Africa is a major challenge especially in the most remote areas. This is especially problematic given the fact that individuals who live in remote areas most often have to travel long distances which means the patient has to pay more out-of-pocket costs to seek medical treatment and care^18^. Other related factors such as the lack of education and health-related knowledge have also been considered risk factors for tuberculosis in South Africa^19^–^20^ together with various other health and lifestyle factors^5^, ^14^. This is especially true for communities such as Mamelodi, a South African township that was developed under apartheid. Apartheid spatial planning laws separated people based on race and resulted in areas that were historically designated as a black African being under-serviced and under-resourced - a problem that continues to the current day.

Although less common, a growing body of research on risk prediction for tuberculosis has identified significant relationships between various built environment factors and tuberculosis 21–23. The notion here is that certain facilities in the built environment may facilitate the spread of the disease either through poor design (i.e., housing^23^) or due to the fact that they promote social gatherings (i.e., churches^22^). Like churches, alcohol outlets are places where people congregate in large numbers over several hours increasing the risk of transmission. The density of alcohol outlets in a neighbourhood has previously been found to be associated with a range of harms including crime^24^, domestic violence^25^, and risky sexual behaviour^26^. Previous public health research has also found that the increased availability of alcohol in an area can lead to a range of other negative physical and mental health outcomes including liver disease^27^, anxiety^28^, injuries^29^, depression^30^ and tuberculosis 21–22, 31. In terms of the latter a study by Munch^21^ found significant associations between tuberculosis and the number of shebeens (informal alcohol outlets) at the neighbourhood level in Cape Town while Murray et al.^22^ also found that shebeens posed a significantly high transmission risk for tuberculosis in Cape Town, particularly if located in overcrowded and impoverished areas of the city. Similar spatial associations between drinking outlets and the risk of tuberculosis infection have been found the United States^8^. While it is increasingly clear that access to alcohol has harmful effects on the population what is less clear is the extent to which geographic access and/or proximity to alcohol outlets is related to tuberculosis at the micro-level. Moreover, most prior research investigating this linkage in South Africa has also been confined to the Western Cape province and has been undertaken at an aggregate level. In this study we aim to employ a number of geospatial techniques to determine the point-based spatial association between alcohol outlet locations and tuberculosis. We also aim to determine whether the underlying socio-demographics of the areas influences this association in any way.

## Methods

Data on the location of tuberculosis incidents in Mamelodi was obtained from the University of Pretoria's (UP) Department of Family Medicine. The Department of Family Medicine obtained point-based tuberculosis information for Mamelodi through their collaboration with the City of Tshwane Metropolitan Municipality's implementation of a Community Orientated Primary Care (COPC) system in the city. The so-called COPC system involves defining geographic areas, otherwise known as wards, and having community health workers collect health-related information pertaining to individuals in each ward with the ultimate aim to use the information obtained to develop and implement community-based health interventions. COPC is a proven approach to primary healthcare that has been successfully implemented throughout the world^32^–^35^. In 2013, the City of Tshwane in collaboration with the Department of Family Medicine at UP initiated COPC by rolling out 23 Ward Based Outreach Teams (WBOT). The first household visits were conducted in August 2014 using a mobile-based health application. In total, the WBOTs collected information of 310,844 individuals across Tshwane including Mamelodi – the study site for this research. The points that fell within the boundaries of Mamelodi were clipped from the total dataset resulting in a total of 115,188 individuals used in the study. After removing null entries and entries which had no GPS coordinates, the total sample of citizen information collected by the WBOTs in Mamelodi was 114,348 individuals. From the survey, three questions were identified as being relevant for the purposes of this research

1. Is anyone in the household currently taking tuberculosis medication?
2. Has anyone in the household been diagnosed with tuberculosis but is not yet taking tuberculosis medicine?
3. Does anyone in the household exhibit symptoms of tuberculosis?

A total of 1742 individuals answered ‘yes’ to any one of these questions. In instances where respondents answered yes to more than one question, only one data point was recorded. The 1742 points were extracted and mapped. Ethical approval for this research was obtained from the University of Pretoria's Faculty of Natural and Agricultural Sciences research ethics committee (approval number EC170612-128).

Data on alcohol outlets in Mamelodi were collected during a number of field trips to the township. An exhaustive data capture exercise resulted in the geo-location of 138 alcohol outlets being captured using a mobile web mapping application. The socio-demographic data used to construct a deprivation index for Mamelodi was obtained from Statistics South Africa. The most recent census for South Africa was undertaken in 2011 and the data recorded in this census was used in the study to construct a deprivation index for the township at the sub-place level of aggregation. This level of aggregation is the finest level at which census data is publicly and exhaustively released; each sub-place consists of between 150 – 300 households.

In this study location quotients (LQs) were employed to compare the extent of tuberculosis around alcohol outlets and their immediate surrounding areas, relative to other areas across the township. Location quotients provide a measure that indicates how different an individual area of interest is relative to the total area under investigation. In the context of this study, a LQ would show the extent to which tuberculosis incidences surrounding an alcohol outlet depart from the overall proportion of tuberculosis occurring throughout Mamelodi as a whole. For example, if an alcohol outlet or the area surrounding an alcohol outlet in Mamelodi has a LQ of 1, then that area has exactly the same relative frequency of tuberculosis as is found across the entire township. If an area surrounding an alcohol outlet has a LQ of 0.5 then that area has half the prevalence of tuberculosis as the overall township and is perhaps a less risky alcohol outlet. Conversely, if an area surrounding an alcohol outlet has a LQ greater than 1, then tuberculosis is over-represented in that alcohol outlet area indicating a relative concentration of tuberculosis at that location. Being a relative measure and without dimension, LQs provide a more vivid measure of risk than for example the high and low indicators of tuberculosis rates. The LQ formula is expressed as follows:

LQGe = (Ge/Ae)/(∑Ge/∑A) (1)

where Ge is the count of tuberculosis locations in each research unit (e.g. alcohol outlet, or buffers around an alcohol outlet), Ae is the area of the corresponding research unit. ∑Ge indicates the total count of tuberculosis incidents in the whole township, and ∑ is the total area of Mamelodi.

In the study, we calculated LQs for a series of buffer intervals immediately surrounding alcohol outlets. This was done in order to determine whether the impact of alcohol outlets on tuberculosis in the areas immediately adjacent to them. We were interested in determining whether individuals that reside closer to an alcohol outlet are at greater risk (spatial diffusion). Buffer intervals of 100 meters, 200 meters and 300 meters were constructed around each alcohol outlet because that distance approximates on average the length of a city block in Mamelodi although these can vary considerably. The density of tuberculosis in these three zones (i.e., 100m; 200m; 300m) are then compared to the density of tuberculosis for the entire township of Mamelodi and represented as a LQ. We also conducted a sensitivity analysis to test our results against the extent of tuberculosis around a randomly selected set of 100 locations throughout the township. For each of these 100 randomly selected point locations, we constructed a 300-meter buffer (broadly approximating a city block in Mamelodi) and calculated LQs for these areas. A comparison of tuberculosis across this ‘control’ group of 100 random locations with the 138 alcohol outlets allows us to be more certain of our findings and provides additional validity to the study.

Finally, we were interested in determining whether the socio-economic characteristics of the neighbourhood in which an alcohol outlet was located was related to the magnitude of tuberculosis in the immediate vicinity of the alcohol outlet. It could be, for instance, that alcohol outlets located in less affluent neighbourhoods are more likely to be have a greater risk of tuberculosis occurrence in surrounding areas and that by aggregating all the alcohol outlets together in our analysis we lose the ability to unmask this association. In order to do this, we constructed a deprivation index using principal components analysis (PCA) and ascribed a deprivation score to each neighbourhood (sub-place) in Mamelodi. The PCA was run on four variables commonly used to measure levels of neighbourhood-level deprivation, namely, 1) the number of people with no income, 2) the number of people who are unemployed, 3) the number of people without grade 12 and above and, 4) the number of people with one room per household.

The PCA identified four components explaining roughly 95% of the variance. The first component explained 94% of the variance and was used as the deprivation score per neighbourhood. LQs for each alcohol outlet were then calculated and averaged per neighbourhood deprivation quintile.

## Results

The results of the LQ analysis are presented in table 1. Overall, the density of tuberculosis around alcohol outlets in Mamelodi at all buffer distances was found to be substantially higher than across the township as a whole (range 1.37–1.47). These findings indicate that the risk of tuberculosis in Mamelodi is higher around alcohol outlets. Interestingly there is no gradient in tuberculosis incidence as the distance from an alcohol outlet increased; in fact, there is a slight decrease in tuberculosis at the 300-meter buffer (LQ = 1.37) compared with the 200-meter buffer (1.47).

**Table 1:** Location quotients of TB around alcohol outlets (n = 138)

Environment	LQ
100m buffer	1.41
200m buffer	1.47
300m buffer	1.37
Random points (100m)	1.05

An examination of the relationship between the socio-economic characteristics of the neighbourhood in which the alcohol outlet was located and LQ of tuberculosis shows no noticeable socio-economic gradient (see Table 2). The average LQ values in quintile 5 (most deprived) were lower than the average LQ values in quintile 4 but greater than the average LQ values in quintile 3 (middle class). Interestingly, the highest average LQ values were found in quintile 2 (affluent).

**Table 2:** Location quotients of tuberculosis around alcohol outlets (n = 138), stratified by deprivation

	Overall	Low				High	Q5:Q1	p-value
		Q1	Q2	Q3	Q4	Q5		
100m buffer	1.41	0.00	2.55	0.99	1.60	1.06	-	0.04*
200m buffer	1.47	0.65	3.38	0.52	1.36	1.05	1.62	0.04*
300m buffer	1.37	0.64	2.52	0.90	1.42	1.16	1.81	0.01*

Note: *p<0.05, **p>0.05

NOTE: There were no incidences of tuberculosis within 100 metres of an alcohol outlet in neighbourhoods in quintile 1 – hence the zero value

## Discussion

The results of our research show that there is a higher density of tuberculosis around alcohol outlets at all buffer distances than Mamelodi as a whole, although there was no monotonic increase nor decrease in tuberculosis as the distance from the alcohol outlet increased. We also found some evidence that the socio-economic background of the neighbourhood in which the alcohol outlet was located significantly associated with occurrence of tuberculosis. Specifically alcohol outlets located in neighbourhoods in quintile 2 exhibited the highest density of tuberculosis on average. It is difficult to compare the results of this research with past research given the limited amount of studies that have examined this association as well as the contextual differences between Mamelodi and other cities. Previous ecological research has however shown that neighbourhoods with higher rates of alcohol outlets consume more alcohol which may increase the risk of tuberculosis transmission^31^, ^36^. This may occur because of increased interaction between people in drinking locations and/or because excessive drinking may lower the body's immune system making infection more susceptible.

One possible reason for the increased density of tuberculosis incidents around alcohol outlets found in our study could be the reciprocal relationship between tuberculosis and alcohol usage. That is, alcohol use increases the risk of contracting tuberculosis, and individuals with tuberculosis disproportionately consume alcohol. This assertion is supported by Peltzeret al^37^ who found that people with tuberculosis were at least 1.3 times more likely to engage in harmful or hazardous drinking. The combination of a highly susceptible individual and an individual with tuberculosis drinking together could potentially explain the spatial association we found. Indeed, a study by Kline et al^8^ found that one index patient with tuberculosis infected 41 of 97 contacts (42 percent) of regular patrons of a neighbourhood bar in Minneapolis.

In our research we also found that the highest mean LQ values were found in more affluent neighbourhoods (quintile 2) when compared to other neighbourhoods. Explanations for this surprising finding are speculative but could be due to ‘pockets of poverty’ located in more affluent neighbourhoods, as found by Erazo et al^40^ or it could be due to the increased mobility in settings such as Mamelodi which have experienced significant inter- and intra-migration since democracy as the associated intermingling of residents across income groups. Past research has found how these types of migration behaviours could increase the risk of tuberculosis^41^,^42^. Overall, however, there were in general higher rates of tuberculosis in alcohol outlets located in poorer neighbourhoods.

This finding is supported past some research which has found an increase in tuberculosis in more deprived neighbourhoods^38^, ^39^. In contrast, in this study however we found the association between tuberculosis and alcohol outlets is more pronounced in more deprived neighbourhoods, adding to the extant literature.

One notable limitation of our study was that we did not take the underlying environmental backcloth into account in our analysis. That is, it could be that the spatial clustering of tuberculosis around alcohol outlets may simply reflect the broader processes (encompassing built, social, cultural and other factors) that are playing out at the neighbourhood level and may not be related to the presence of alcohol outlets themselves. This concern has validity, however, much of the appeal of location quotients lies in its simplicity. The technique is not analytical but is a purely descriptive measure which allows users to determine more broadly where a phenomenon is spatially clustered. Using this technique we found that the density of tuberculosis is greater in the areas surrounding alcohol outlets when compared to Mamelodi as a whole. Future research can aim to incorporate additional confounders into subsequent analysis to supplement the work done in this study.

## Conclusion

This study represents the first empirical attempt to investigate the spatial association between tuberculosis and alcohol outlets at the point-based level in South Africa. Previous literature has examined tuberculosis prevalence either at the aggregate level or examined the phenomenon in association to a number of places where individuals congregate such as community halls, or churches. Alcohol outlets in Mamelodi form an integral part of the informal economy and are an essential means by which a number of households earn a living. They are therefore a permanent and necessary feature of the urban landscape. The results of our research however indicate that they may facilitate the transmission of tuberculosis, at least in Mamelodi, which may be exacerbated in more deprived neighbourhoods. Further research could aim to investigate whether it is the mere purchase of alcohol from outlets or the consumption of alcohol at these outlets that increase the spatial risk. Studies in other contexts both in South Africa specifically, and Africa more generally, would also be of value.

## References

[1] Annabel B, Anna D, Hannah MD (2019). Global tuberculosis report 2019.

[2] Coetzee N, Yach D, Joubert G (1988). Crowding and alcohol abuse as risk factors for tuberculosis in the Mamre population. Results of a case-control study. S African Med J.

[3] Dudley L, Mukinda F, Dyers R, Marais F, Sissolak D (2018). Mind the gap! Risk factors for poor continuity of care of TB patients discharged from a hospital in the Western Cape, South Africa. PLoS One.

[4] Jordan AM, Podewils LJ, Castro KG, Zishiri V, Charalambous S (2019). Prevalence and risk factors of tuberculosis disease in South African correctional facilities in 2015. Int J Tuberc Lung Dis.

[5] Mahtab S, Coetzee D (2017). Influence of HIV and other risk factors on tuberculosis. S African Med J.

[6] Tadokera R, Bekker L, Kreiswirth BN (2020). TB transmission is associated with prolonged stay in a low socio-economic, high burdened TB and HIV community in Cape Town, South Africa. BMC Infect Dis.

[7] vanRie A, McCarthy K, Scott L, Dow A, Venter WD, Stevens WS (2013). Prevalence, risk factors and risk perception of tuberculosis infection among medical students and healthcare workers in Johannesburg, South Africa. S Afr Med J.

[8] Kline SE, Hedemark LL, Davies SF (1995). Outbreak of tuberculosis among regular patrons of a neighbourhood bar. N Engl J Med.

[9] Simou E, Britton J, Leonardi-Bee J (2018). Alcohol and the risk of pneumonia: a systematic review and meta-analysis. BMJ Open.

[10] Antunes JLF, Waldman AE (2001). The impact of AIDS, immigration and housing overcrowding on tuberculosis deaths in Sao Paulo, Brazil, 1994–1998. SocSci Med.

[11] Bhunu CP, Mushayabasa S, Smith RJ (2012). Assessing the effects of poverty in tuberculosis dynamics. Appl Math Model.

[12] Cramm JM, Koolman X, Moller V, Nieboer AP (2011). Socio-economic status and self-reported tuberculosis: a multilevel analysis in a low-income township of Easter Cape, South Africa. J of Public Health in Africa.

[13] Bruce N, Perez-Padilla R, Albalak R (2000). Indoor air pollution in developing countries: a major environmental and public health challenge. Bull World Health Organ.

[14] Brunet L, Pai M, Davids V, Ling D, Paradis G, Lenders L (2011). High prevalence of smoking among patients with suspected tuberculosis in South Africa. EurRespir J.

[15] Erlinger S, Stracker N, Hanrahan C (2019). Tuberculosis patients with higher levels of poverty face equal or greater costs of illness. Int J Tuberc Lung Dis.

[16] Harling G, Ehrlich R, Myer L (2008). The social epidemiology of tuberculosis in South Africa: a multilevel analysis. SocSci Med.

[17] Chimbindi NZ, Barnighausen T, Newell ML (2013). An integrated approach to improving the availability and utilisation of tuberculosis healthcare in rural South Africa. S Afr Med J.

[18] World Health Organization (2005). Global tuberculosis control: surveillance, planning, financing.

[19] Kigozi NG, Heunis JC, Engelbrecht MC (2017). Tuberculosis knowledge, attitudes and practices of patients at primary health care facilities in a South African metropolitan: research towards improved health education. BMC Public Health.

[20] Kootbodien T, Wilson K, Tlotleng N (2018). Tuberculosis mortality by occupation in South Africa, 2011–2015. Int J Environ Res Public Health.

[21] Munch Z, Van Lill SWP, Booysen CN (2003). Tuberculosis transmission patterns in a high-incidence area: a spatial analysis. Int J Tuberc Lung Dis.

[22] Murray EJ, Marais BJ, Mans G, Beyers N, Ayles H, Godfrey-Faussett P (2009). A multidisciplinary method to map potential tuberculosis transmission ‘hot spots' in high-burden communities. Int J Tuberc Lung Dis.

[23] Nasir Z, Margaritis A, Lai KM, Colbeck I, Chatterjee A Impact of built environment characteristics on risk of TB transmission in slums.

[24] Grubesic TH, Pridemore WA (2011). Alcohol outlets and clusters of violence. Int Journal of Health Geographics.

[25] Livingston M (2011). A longitudinal analysis of alcohol outlet density and domestic violence. Addiction.

[26] Letsela LR, Weiner M, Gafos, Fritz K (2019). Alcohol availability, marketing, and sexual health risk amongst urban and rural youth in South Africa. AIDS Behav.

[27] Major JM, Sargent JD, Graubard BI, Carlos HA, Hollenbeck AR, Altekruse SF (2014). Local geographic variation in chronic liver disease and hepatocellular carcinoma: contributions of socioeconomic deprivation, alcohol retail outlets, and lifestyle. Ann Epidemiology.

[28] Pereira G, Wood L, Foster S, Haggar F (2013). Access to alcohol outlets, alcohol consumption and mental health. PLoS One.

[29] Morrison C, Smith K, Gruenewald PJ, Ponicki WR, Lee JP, Cameron P (2016). Relating off-premises alcohol outlet density to intentional and unintentional injuries. Addiction.

[30] Lamb KE, Thornton LE, Teychenne M, Milte C, Cerin E, Ball K (2017). Associations between access to alcohol outlets and alcohol intake and depressive symptoms in women from socioeconomically disadvantaged neighbourhoods in Australia. BMC Public Health.

[31] Classen CN, Warren R, Richardson M, Hauman JH, Gie RP Impact of social interactions in the community on the transmission of tuberculosis in a high incidence area. Thorax.

[32] Kinkel HF, Marcus TS, Memon S, Bam N, Hugo JFM (2012). Community oriented primary care in Tshwane District, South Africa: assessing the first phase of implementation. Afr J Prim Health Care Fam Med.

[33] Longlett SK, Kruse JE, Wesley RM (2001). Community-oriented primary care: historical perspective. J Am Board FamPract.

[34] Mettee TM, Martin KB, Williams RL (1998). Tools for community-oriented primary care: a process for linking practice and community data. J Am Board FamPract.

[35] Meyer ED, Hugo JFM, Marcus TS, Molebatsi R, Komana K (2018). Why high tech needs high touch: Supporting continuity of community primary healthcare. Afr J Prim Health Care Fam Med.

[36] Young R, Macdonald L, Ellaway A (2013). Associations between proximity and density of local alcohol outlets and alcohol use among Scottish adolescents. Health and Place.

[37] Peltzer K, Louw J, Mchunu G, Naidoo P, Matseke G, Tutshana B (2012). Hazardous and harmful alcohol use and associated factors in tuberculosis public primary care patients in South Africa. Int J Environ Res Public Health.

[38] Souza WV, Ximenes R, Albuquerque MFM, Lapa TM, Portugal JL, Lima ML (2000). The use of socioeconomic factors in mapping tuberculosis risk areas in a city of northeastern Brazil. Pan Am J Public Health.

[39] Santos MLSG, Vendramini SHF, Gazetta CE, Oliveira SAC, Villa TCS (2007). Poverty: socioeconomic characterization at tuberculosis. Rev Latino-am Enfermagem.

[40] Erazo C, Pereira SM, Costa MCN, Evangelista-Filho D, Braga JU, Barreto ML (2014). Tuberculosis and living conditions in Salvador, Brazil: a spatial analysis. Rev Panam-Salud-Publica.

[41] Wood R, Racow K, Bekker L, Morrow C, Middelkoop K, Mark D, Lawn SD (2012). Indoor social networks in a South African township: Potential contribution of location to tuberculosis transmission. PLoS One.

[42] Lima SVMA, dos Santos AD, Duque AM, Goes MA (2019). Spatial and temporal analysis of tuberculosis in an area of social inequality in Northeast Brazil. BMC Public Health.

